# Genetic and physiological characteristics of *CsNPR3* edited citrus and their impact on HLB tolerance

**DOI:** 10.3389/fgeed.2024.1485529

**Published:** 2024-12-04

**Authors:** Trishna Tiwari, Cecile Robertson, Choaa El-Mohtar, Jude Grosser, Tripti Vashisth, Zhonglin Mou, Manjul Dutt

**Affiliations:** ^1^ Department of Horticultural Sciences, Citrus Research and Education Center, University of Florida, Lake Alfred, FL, United States; ^2^ Plant Breeding Graduate Program, University of Florida, Gainesville, FL, United States; ^3^ Department of Plant Pathology, Citrus Research and Education Center, University of Florida, Lake Alfred, FL, United States; ^4^ Department of Microbiology and Cell Science, University of Florida, Gainesville, FL, United States

**Keywords:** citrus, HLB, genome editing, transgenics, SAR

## Abstract

Huanglongbing (HLB) disease, caused by *Candidatus* Liberibacte*r* asiaticus (*Ca*Las), severely impacts citrus production, and currently, there is no cure. Developing HLB-resistant or tolerant cultivars is crucial, with modifying defense-related genes being a promising approach to managing HLB. NONEXPRESSOR OF PATHOGENESIS-RELATED GENES 1 (NPR1) is a positive regulator of systemic acquired resistance (SAR), which enhances resistance to pathogens, whereas NONEXPRESSOR OF PATHOGENESIS-RELATED GENES 3 (NPR3) is a negative regulator of SAR. To unambiguously address the role of *CsNPR3* in HLB, we introduced mutations into the *CsNPR3* gene in sweet orange (*Citrus sinensis* L. Osbeck) through genome editing and assessed their effects on morphology, physiology, and resistance/tolerance to HLB. Several genome-edited ‘Hamlin’ sweet orange trees harboring frameshift-inducing insertions or deletions were identified. After confirming the genome editing using Sanger sequencing, selected lines were grafted onto C-146 trifoliate hybrid rootstocks for clonal propagation. The progenies were then infected with *Ca*Las using a no-choice Asian Citrus Psyllid (ACP) feeding assay. Evaluation of the genetic and physiological characteristics of *CsNPR3*-edited citrus trees under greenhouse conditions revealed that the edited trees exhibited greater vigor than the wild-type trees, despite the lack of significant differences in *Ca*Las titers. Although further field evaluation is needed, our findings indicate that *CsNPR3* contributes to HLB-caused tree deterioration and demonstrate that editing *CsNPR3* can enhance tolerance to HLB.

## Introduction

Huanglongbing (HLB), also known as citrus greening, caused by *Candidatus* Liberibacter asiaticus (*Ca*Las), is a severe threat to citrus production worldwide, with no known cure to date (Huang et al., 2021). First reported in the USA in 2005, HLB severely affected all citrus-growing areas in Florida, the state with the largest citrus industry (Wang and Trivedi, 2013). HLB is transmitted by an insect vector, the Asian citrus psyllid [(ACP; *Diaphorina citri* (Kuwayama)] (Ammar et al., 2020). HLB symptoms include asymmetrical yellowing of leaves; distorted foliage; small, green, and misshapen fruits; aborted seeds; bitter juice; twig dieback; yield reduction; and tree decline (Gottwald et al., 2007). In addition to morphological symptoms, HLB induces several physiological and chemical changes. For instance, HLB increases the starch accumulation and enlargement of starch granules in the leaves of the infected trees compared with those in the leaves of uninfected trees (Etxeberria et al., 2009). Sucrose, glucose, and fructose levels have also been shown to be significantly elevated in infected trees (Meng et al., 2019). Furthermore, *Ca*Las infection activates chlorophyll-degrading enzymes, leading to chlorophyll degradation in HLB-infected leaves (Cai et al., 2022). The severity and progression of symptoms may vary; however, once a plant is infected, the yield and quality decrease gradually, ultimately leading to its death (Gottwald, 2010).

Current HLB management practices encompass various methods, including cultural practices, chemical control, biocontrol, breeding approaches, and systemic acquired resistance (SAR; Li et al., 2020). Cultural practices aim to maintain tree health and vigor through proper irrigation and fertilization and using disease-free planting materials. Protected growing systems such as Citrus Under Protective Screen (CUPS) have also been employed to mitigate disease spread. Chemical and biocontrol strategies involve the use of insecticides or biocontrol agents to manage disease-spreading insect vectors. Breeding efforts focus on developing citrus cultivars resistant or tolerant to HLB. Targeting SAR pathways also enhances HLB resistance (Gottwald, 2010; Dala-Paula et al., 2019; Li et al., 2021; Schumann et al., 2022). Among these strategies, developing disease-resistant/tolerant cultivars using different breeding techniques is an effective approach to managing HLB by preventing tree deterioration (Qureshi et al., 2014), with increased yield and improved fruit quality.

Modern genome editing techniques like Clustered Regularly Interspaced Short Palindromic Repeats (CRISPR)/CRISPR-associated protein 9 (Cas9) allow precise genetic modifications in trees to offer resistance against major pathogens. For instance, CRISPR/Cas9 has been used to successfully edit the *phytoene desaturase* (*PDS*) gene in apples (Nishitani et al., 2016), citrus (Jia et al., 2017), grapes (Nakajima et al., 2017), and strawberries (Wilson et al., 2019), and various other genes in several other crops. CRISPR/Cas9 also allows simultaneous targeting of multiple genes (Chen et al., 2019). However, the potency of CRISPR/Cas9 in promoting plant defense responses against *Ca*Las infection has not been explored.

SAR is a plant defense mechanism induced by mobile signals produced at the infection site (Durrant and Dong, 2004; Dutt et al., 2015). Upon pathogen infection, salicylic acid (SA) accumulates both locally and systemically to prevent further infection by controlling the onset of local and systemic acquired resistance (Gao et al., 2015). SA production after a pathogen attack triggers the expression of pathogenesis-related (PR) proteins (Mou et al., 2003; Dutt et al., 2015). The NONEXPRESSOR OF PATHOGENESIS-RELATED GENES 1 (*NPR1*) is a SA receptor that plays a central role in regulating the expression of PR proteins involved in various defense responses against invading pathogens (Maier et al., 2010; Ding et al., 2018). Briefly, upon pathogen infection, NPR1 is reduced to its monomeric form and translocated from the cytoplasm to the nucleus, where it interacts with TGA transcription factors, resulting in *PR* gene induction (Shi et al., 2010). Overexpression of the *Arabidopsis* derived *AtNPR1* has been shown to enhance plant disease resistance in many crops, including apple (Malnoy et al., 2007), citrus (Dutt et al., 2015; Robertson et al., 2018), rice (Chern et al., 2001), tobacco (Zhang et al., 2010), tomato (Lin et al., 2004) and wheat (Makandar et al., 2006).

Two paralogs of *NPR1*, namely, NONEXPRESSOR OF PATHOGENESIS-RELATED GENES 3 (*NPR3)* and NONEXPRESSOR OF PATHOGENESIS-RELATED GENES 4 (*NPR4)*, bind SA and function as transcriptional co-repressors during plant defense against pathogens (Ding et al., 2018). They also function as SA-regulated adaptors of the Cullin 3 ubiquitin E3 ligase, facilitating the degradation of *NPR1* (Fu et al., 2012; Ding et al., 2016). Nevertheless, these three SA receptors play opposing roles in the transcriptional regulation of SA-induced defense genes (Ding et al., 2018). In the absence of pathogen infection or low levels of SA, NPR3/NPR4 represses the expression of defense-related genes, thereby preventing autoimmunity (Ding et al., 2018). Loss of *NPR3* and *NPR4* has been shown to elevate the expression of *PR* genes and enhance disease resistance in the *npr3 npr4* double mutants in *Arabidopsis* (Zhang et al., 2006). Moreover, knockdown of the *NPR3* ortholog in *Theobroma cacao* (*TcNPR3*) decreases susceptibility to *Phytophthora capsici* likely through elevated *PR* expression (Shi et al., 2013).

In this study, we developed *CsNPR3*-edited sweet orange lines to clearly determine the function of *CsNPR3* in HLB disease. Genetic and physiological characterization of the *CsNPR3*-edited trees demonstrated that *CsNPR3* significantly contributes to HLB-caused tree deterioration. These findings suggest that editing *CsNPR3* is a promising strategy for enhancing HLB tolerance in susceptible citrus varieties.

## Materials and methods

### Development of genome edited hamlin sweet orange lines using CRISPR/Cas9

**FIGURE 1:**
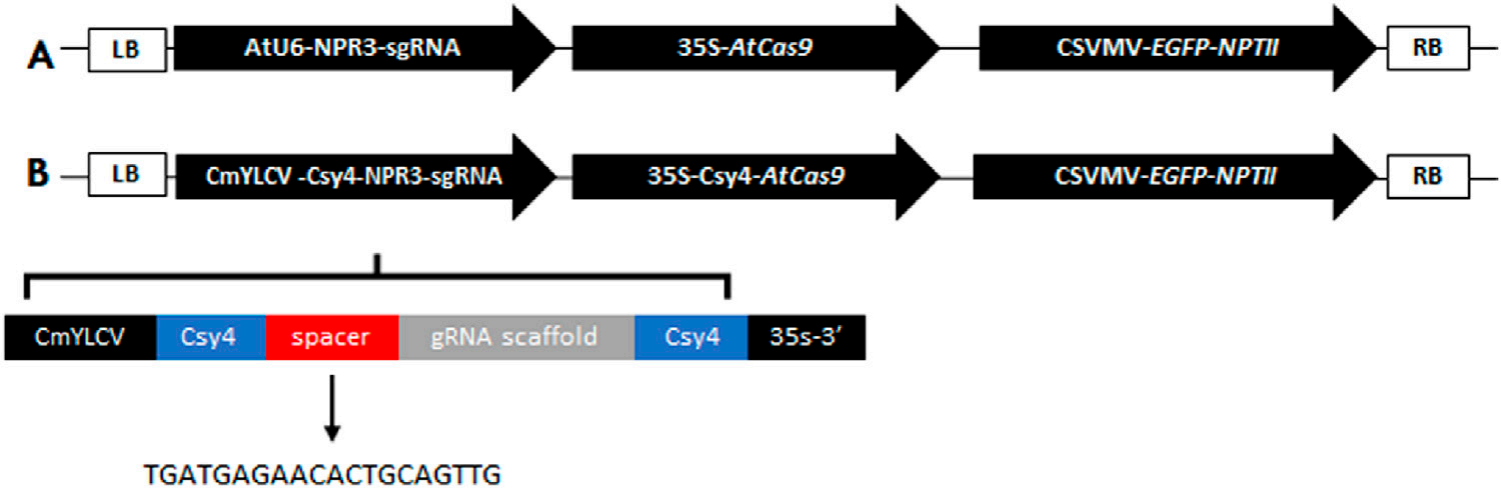
DNA constructs used in the study **(A)** Construct containing a 35S promoter driven AtCas9 gene. The Arabidopsis U6-26 promoter was utilized to drive the expression of the gRNAs in this vector. **(B)** Construct containing a 35S promoter-driven *AtCas9* gene fused to the Csy4 bacterial endoribonuclease from *Pseudomonas aeruginosa* (Csy4-Cas9). The Cestrum yellow leaf curling virus (CmYLCV) promoter was fused to the Csy4 recognition sequence to release mature gRNA in this vector.

In this study, we generated Hamlin (*Citrus sinensis* L. Osbeck) sweet orange lines through CRISPR/Cas9-mediated editing of the *CsNPR3* coding region. Briefly, the guide RNA (gRNA) specific to *CsNPR3* was designed using the genomic DNA (orange1.1g007849 m.g; Phytozome database; https://phytozome-next.jgi.doe.gov/) and the CRISPRdirect software (https://crispr.dbcls.jp/; Naito et al., 2015). This gRNA (5′ TGA​TGA​GAA​CAC​TGC​AGT​TG 3′) targeted the second exon of the *CsNPR3* gene. For the genetic transformation of citrus (Dutt and Grosser, 2009), we developed two DNA constructs that were based on previous constructions (Dutt et al., 2020): the first one contained a 35S promoter-driven *AtCas9*, with the gRNA expression under the control of Arabidopsis U6-26 promoter. All transgenic lines produced with this construct have the prefix ‘C’. The second construct contained the 35S promoter-driven *AtCas9* gene fused in frame to the Csy4 bacterial endoribonuclease from *Pseudomonas aeruginosa* (Csy4-Cas9). The expression of sgRNAs processed by Csy4 to release the sgRNAs was driven by the CmYLCV promoter; all transgenic lines produced with this construct have the prefix ‘CC’ (Figure 1). Transgenic lines were selected based on the EGFP expression (Supplementary Figure S1). OneTaq^®^ Hot Start 2X Master Mix with standard buffer (New England Biolabs, Ipswich, MA, USA) was used for PCR, and the products were sequenced directly using the Sanger method. The sequencing results were compared with the sequence of the *CsNPR3* gene by alignment using SnapGene software. The selected genome-edited lines were clonally propagated on a C-146 trifoliate hybrid rootstock (*Citrus sunki* Hort. Ex Tan. × *Poncirus trifoliata* L. Raf. cv. Swingle). Six months old, budded trees were infected with *Ca*Las using HLB-infected free-flying psyllids in a growth chamber. Infected trees were maintained in an air-conditioned greenhouse (77°F/25°C) at the University of Florida’s Citrus Research and Education Center (Lake Alfred, FL). Leaf samples were collected at different time points for further analysis.

### *Ca*Las infection and evaluation

The clonally propagated genome-edited lines were infected with *Ca*Las using a no-choice ACP feeding assay. To determine the titer of *Ca*Las in the leaves, genomic DNA was extracted from the petioles and midveins of fully expanded leaves at 6, 12, 18, and 24 months after infection. DNA extraction was carried out using the GeneJET Plant Genomic DNA Purification Kit (Thermo Fisher Scientific) according to the manufacturer’s protocol. The extracted DNA was then normalized to 25 ng/μL prior to quantitative PCR (qPCR) analysis. qPCR was performed using a StepOnePlus™ Real-Time PCR System (Thermo Fisher Scientific). *Ca*Las DNA was detected using TaqMan™ Gene Expression Master Mix and a primer and probe set targeting a segment of the rplJ/rplL (Wang et al., 2006). Samples with Ct values greater than 36 were regarded as negative, while those with Ct values equal to or less than 32 were considered positive.

### RNA extraction and cDNA synthesis

RNA was extracted from the leaf samples using the Direct-zol™ RNA Miniprep Plus Kit (Zymo Research, CA, USA). RNA concentration was determined using a NanoDrop™ 1,000 spectrophotometer (Thermo Fisher Scientific, Franklin, MA, USA). The purity and integrity of the RNA were analyzed by electrophoresis on a 1.0% agarose gel using an Agilent 2,100 Bioanalyzer (Agilent Technologies, Santa Clara, CA). High-quality RNA was used for cDNA synthesis. Single-stranded cDNA was synthesized by reverse transcription using a PrimerScript™ RT Reagent Kit (TaKara Bio USA, Inc., San Jose, CA) following the manufacturer’s protocol.

### Gene expression using qPCR

Quantitative polymerase chain reaction (qPCR) was performed using SYBR^®^ Green PCR Master Mix (Applied Biosystems, Foster City, CA) coupled with different primers. The qPCR mixture comprised 1 μL diluted cDNA (40 ng/μL), SYBR^®^ Green PCR Master Mix, qPCR water, and primers (forward and reverse). The reaction was carried out in a total volume of 10 μL according to the manufacturer’s instructions. Differential gene expression analysis was conducted on the genes listed in Supplementary Table S1 using the SYBR Green assay. The relative expression of the target gene was determined using the ΔΔCt method, with the β-actin gene serving as an internal housekeeping control for all qPCR experiments.

### Plant growth parameters

Plant growth parameters such as plant height, leaf area, number of leaves, and trunk diameter were measured at the end of the experiment. Destructive sampling was performed to measure the total fresh weight of the trees. A well-calibrated Vernier-caliper was used to determine the trunk diameter. Tree height was measured using a metric ruler, with the graft union serving as the reference point. To calculate leaf area, ten fully expanded mature leaves were randomly selected, scanned using a flatbed scanner, and analyzed using ImageJ software.

### Determination of chlorophyll pigment content

Total chlorophyll content was estimated using the Soil Plant Analysis Development (SPAD) index (502DL Plus chlorophyll meter, Spectrum Technologies, Inc., Aurora, IL, USA) on plant leaves under protected conditions. SPAD readings of ten fully expanded mature leaves were obtained at three locations. The SPAD values for each tree were averaged and the results were expressed as the chlorophyll content index (CCI). Chlorophyll a, chlorophyll b, total chlorophyll, and total carotenoids were extracted using 96% (v/v) ethanol, according to the method described by Lichtenthaler and Buschmann (2001). The solutions were stored in the dark for 24 h to ensure complete extraction of chlorophyll and later measured using GENESYS™ 30 Visible Spectrophotometer (Thermo Fisher Scientific Inc.) at different wavelengths of 470, 665, and 649 nm.

### Determination of starch content

The starch content in the leaves was analyzed as described by Gonzalez et al. (2012), with slight modifications. Briefly, the leaves were ground to powder and homogenized in 700 μL distilled water. The leaf samples and standard (rice starch) were boiled in a water bath for 10 min. The samples were then cooled, vortexed, and centrifuged for 2 min at 6,000 rpm. The supernatant (approximately 300 μL) was extracted with 900 μL absolute alcohol. The mixture was then vortexed and centrifuged for 10 min at 10,000 rpm. The resulting supernatant was discarded, and 1 mL distilled water was added to dissolve the pellet, followed by the addition of 50 μL KI: I2 (8:50 mM). The color change was monitored in the GENESYS™ 30 Visible Spectrophotometer (Thermo Fisher Scientific Inc.) at 594 nm (Mahmoud et al., 2021).

### Determination of total phenolic content

Total phenolic content (TPC) was estimated using total phenolic content assay according to the protocol described by Sánchez-Rangel et al. (2013). TPC was extracted in 1 mL ethanol and centrifuged at 12,000 rpm for 10 min at 20°C, followed by the addition of sodium carbonate (Na_2_CO_3_) at 7.5% (*w/v*). The reaction mixture was then incubated at 25°C for 1 h. Different concentrations of aqueous gallic acid solutions from 100 to 600 were used as standard solutions. Absorbance was recorded at 765 nm in GENESYS™ 30 Visible Spectrophotometer (Thermo Fisher Scientific Inc.). The results are expressed as mg gallic acid (GAE) g^−1^ fresh weight (FW).

### Determination of DPPH radical-scavenging activity

The 2,2-diphenyl-1-picryl-hydrazyl-hydrate (DPPH) radical-scavenging activity was determined using the DPPH assay, as described in a previous study (Sharma and Bhat, 2009). Briefly, equivalent amounts of DPPH solution and plant extract were mixed and incubated for 30 min. The absorbance was recorded at 517 nm in GENESYS™ 30 Visible Spectrophotometer (Thermo Fisher Scientific Inc.) with methanol as the blank solution. The control solution was prepared by adding DPPH to methanol. The following equation was used to calculate the percentage of DPPH inhibition:

$$\%\text{ DPPH inhibition}=\frac{\left(A_{Control}-A_{Sample}\right)}{\left(A_{Control}\right)}\times 100$$

where $A_{Control}$ is the absorbance of the control $A_{Sample}$ is the absorbance of the extract with the DPPH solution.

### Statistical analysis

Statistical analysis was conducted using analysis of variance (ANOVA). For mean comparisons between treatments, the Fisher Least Significant Difference (LSD) test was employed with a significance level set at *p* < 0.05. Each treatment included five to six biological replicates. All statistical analyses were performed using RStudio software.

## Results

### Sanger sequencing confirmed the gene-edited lines

**FIGURE 2:**
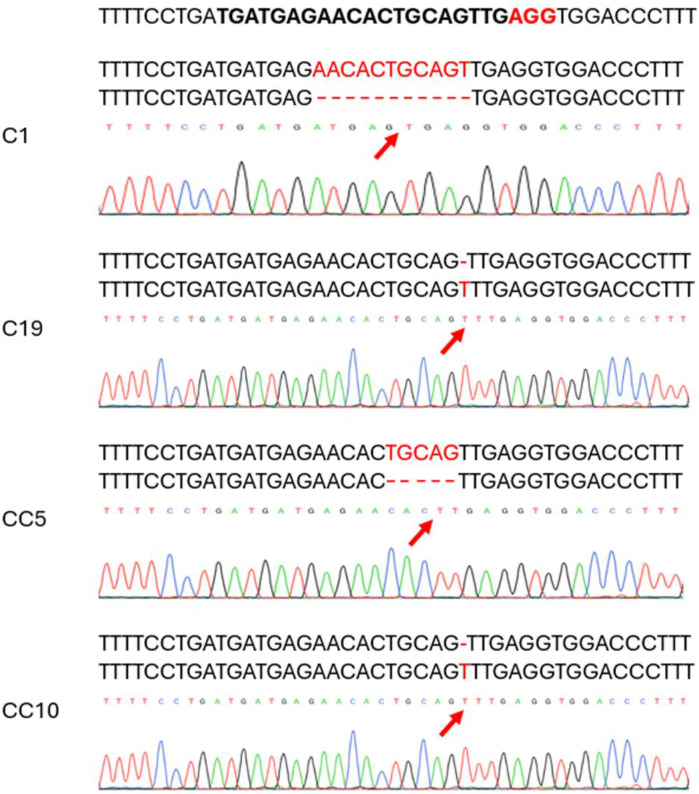
Chromatograms showing mutation types of selected edited lines as revealed by Sanger sequencing on the Cas9 edited lines. The top sequence is the target specific crRNA sequence in bold with the protospacer adjacent motif (PAM) in red. The mutated sequence is aligned with the wild type on top of each chromatogram. Red arrow indicates the deletion/base insertion site.

We generated 21 lines in this study. A preliminary Sanger sequencing with the purified PCR product identified mutations in several lines. Cloned PCR products from several of the potential lines were sequenced to confirm the mutations in these putative edited lines and to identify mutations. Frameshift mutations were identified after analyzing the lines using the SnapGene software. Edited lines were selected based on their overall vigor and edits. Two selected edited lines carry deletions: line C1 had a deletion of 11 bases, and line CC5 had a deletion of 5 bases. In addition, two other edited lines, C19 and CC10, each had an insertion of a single base (Figure 2).

### *CsNPR3* editing altered the expression of *PR* transcripts

**FIGURE 3:**
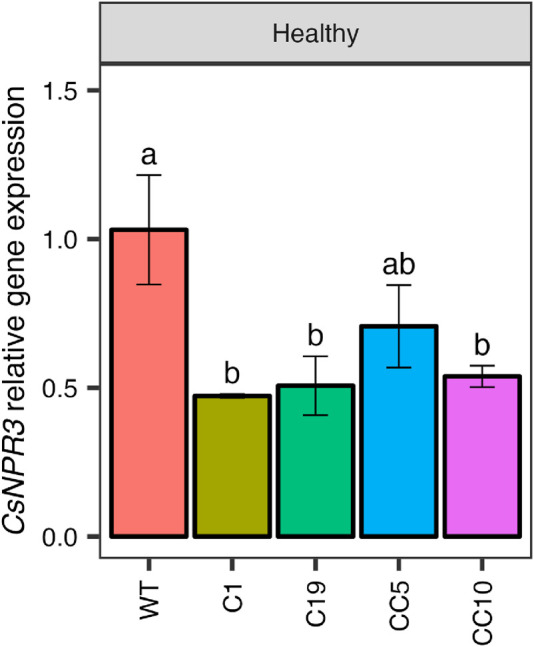
*CsNPR3* expression levels in the genome edited lines.

The 4 selected genome edited lines were clonally propagated and 6 trees from each line were used in this study. The relative expression of *CsNPR1*, *CsNPR3*, *CsNPR4*, and several *PR* genes (*CsPR1*, *CsPR2*, and *CsPR5*) were analyzed initially in the clonally propagated *CsNPR3*-edited and non-edited (WT) trees. The qPCR results indicated a significant decrease in *CsNPR3* transcript levels across all edited lines compared to those in wild-type trees (Figure 3).

**FIGURE 4:**
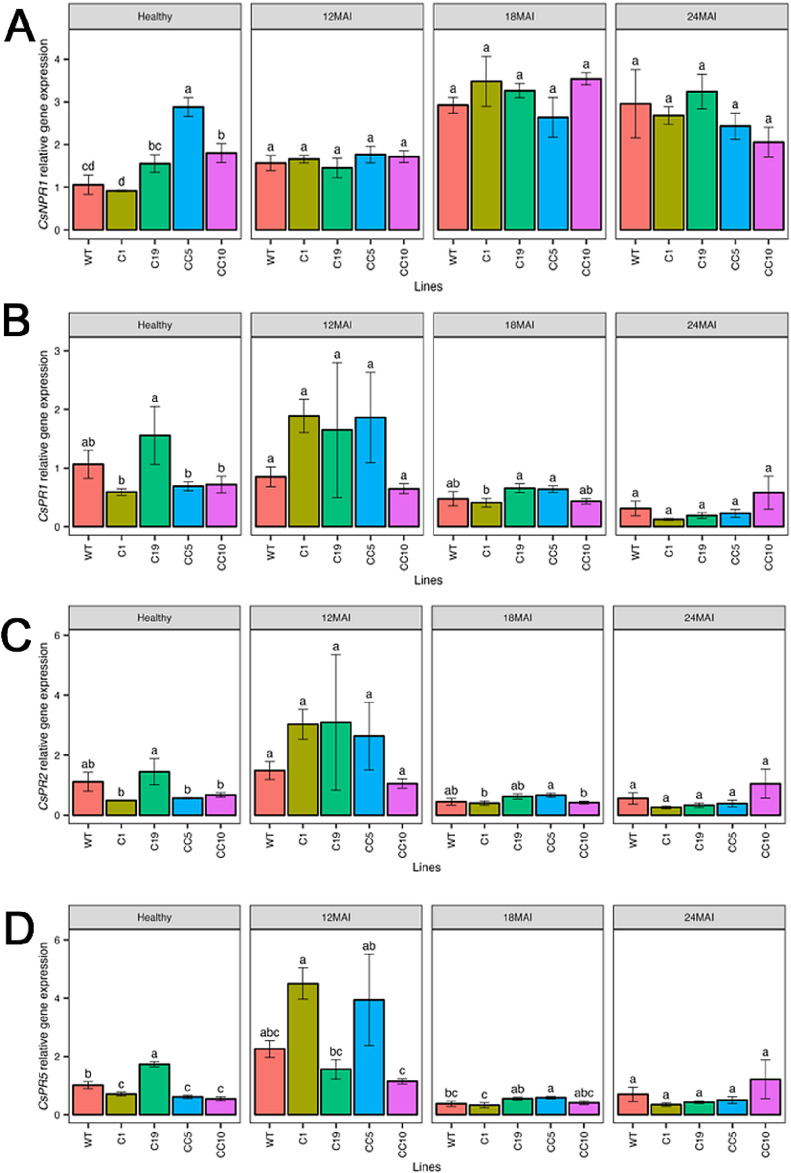
Relative gene expression level of *CsNPR1* **(A)**, *CsPR1* **(B)**, *CsPR2* **(C)** and *CsPR5* **(D)** in the edited lines and wild type. Analysis of Variance (ANOVA) was carried out separately for different times (12 MAI - 12 months after infection, 18 MAI- 18 months after infection, 24 MAI- 24 months after infection); WT- Wild Type. Different letters on bars indicate statistically significant differences among lines by Fisher’s LSD at *p* < 0.05). Error bar indicates standard error.

**FIGURE 5:**
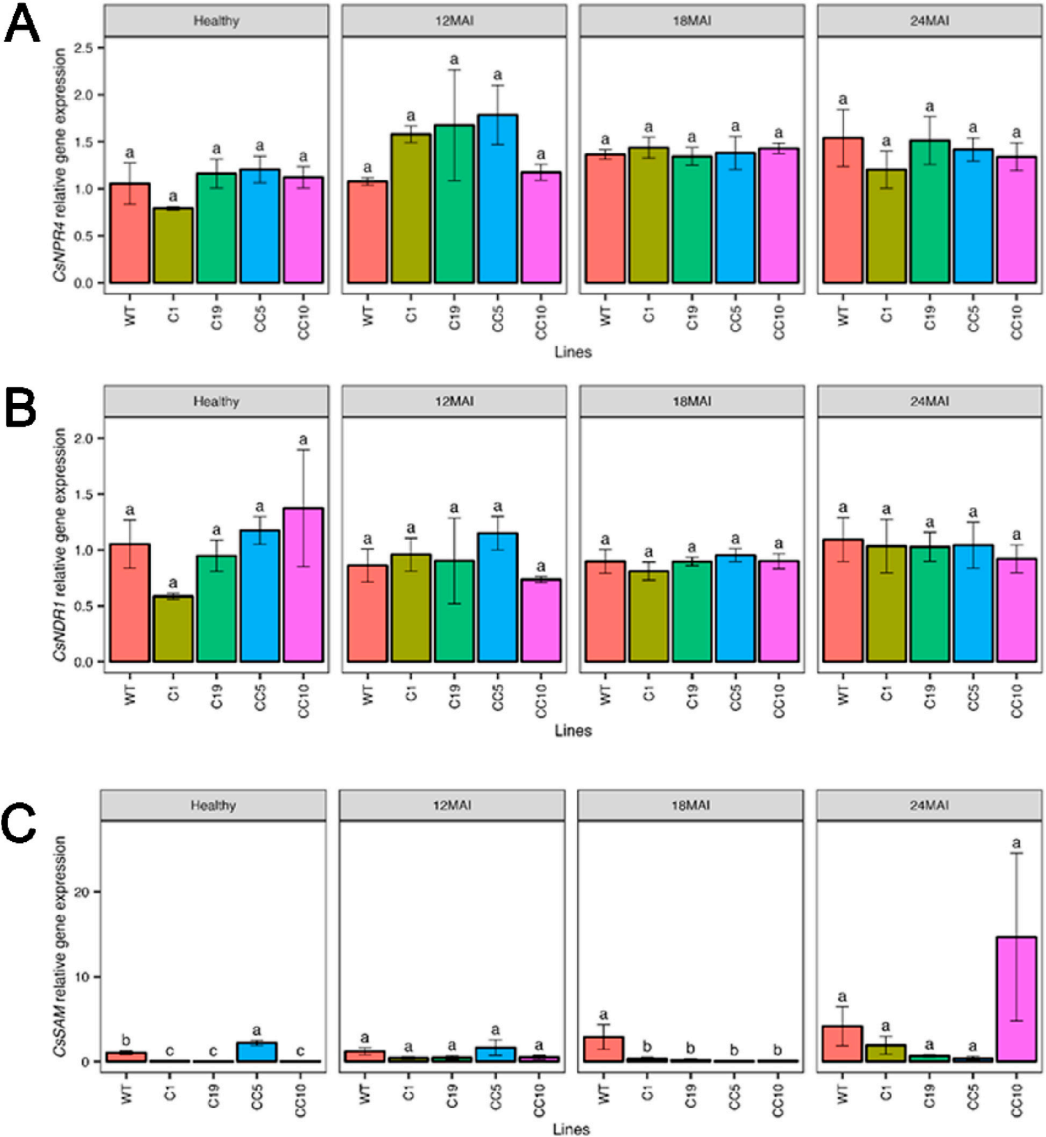
Relative gene expression level of *CsNPR4* **(A)**, *CsNDR1* **(B)** and *CsSAM* **(C)** in the edited lines and wild type. Analysis of Variance (ANOVA) was carried out separately for different times (12 MAI - 12 months after infection, 18 MAI- 18 months after infection, 24 MAI- 24 months after infection); WT- Wild Type. Different letters on bars indicate statistically significant differences among lines by Fisher’s LSD at *p* < 0.05). Error bar indicates standard error.

The relative expression levels of *CsNPR1* in the edited lines were, however, similar to those in the WT lines, except in line CC5, which exhibited significantly higher *CsNPR1* transcript levels than those in the WT and all other edited lines (*p* < 0.001; Figure 4A). The relative expression of *CsPR1* and *CsPR2* did not differ significantly from those in the WT and any of the edited lines (Figures 4B, C). In contrast, expression of *CsPR5* was significantly elevated in some of the edited lines compared to that in the WT, after 12 months following infection (*p* < 0.001; Figure 4D). The relative expression of *CsNPR4* (Figure 5A) and non-race-specific disease resistance 1 in citrus (*CsNDR1;* Figure 5B) did not display significant differences between the edited lines and the WT trees (*p* = 0.34 and *p* = 0.36, respectively). There was a significant difference (*p* < 0.0001) in *CsSAM* expression among the edited lines, with line CC5 showing the highest expression compared to all other lines (*p* < 0.001; Figure 5C).

After infection with *Ca*Las, analysis of gene expression changes in infected edited lines and WT revealed that *CsNPR3* remained downregulated in all edited trees compared to the control specifically after 24 months of infection. In contrast, the expression level of *CsNPR1*, *CsPR1, CsPR2, Cs PR5, CsNPR4,* and *CsNDR1* did not differ significantly between the edited and the WT trees throughout the study period.

### Susceptibility of *CsNPR3* edited lines to HLB

The edited and WT lines were then infected with *Ca*Las in a 3-month no-choice feeding assay using infected psyllids. Leaves were sampled at 6, 12, 18, and 24 months post-infection and *Ca*Las bacterial titers were quantified from leaf petioles and midribs using qPCR. All lines used in this study (control and gene-edited) tested positive for *Ca*Las 6 months after infection and remained positive throughout the study (Table 1).

**TABLE 1:** Quantification of *Ca*Las bacterial titers following qPCR from leaf petiole and midribs of the transgenic trees and controls grown under greenhouse conditions and exposed to free-flying, potentially *Ca*Las-positive psyllids. The mean threshold cycle values (Ct) at specified time intervals are indicated.

Time after infection	Control	Transgenic line C1	Transgenic line C19	Transgenic line CC5	Transgenic line CC10
6 months	24.73 ± 0.15	24.36 ± 0.15	26.21 ± 0.5	25.16 ± 0.11	24.34 ± 0.04
12 months	21.85 ± 0.08	21.99 ± 0.13	21.44 ± 0.03	21.16 ± 0.03	22.28 ± 0.08
18 months	23.51 ± 0.26	25.98 ± 0.61	23.47 ± 0.19	22.51 ± 0.14	22.54 ± 0.11
24 months	23.71 ± 0.28	24.47 ± 0.15	24.37 ± 0.24	23.83 ± 0.11	24.64 ± 0.29

### Gene-edited trees exhibited enhanced growth compared to WT trees

**FIGURE 6:**
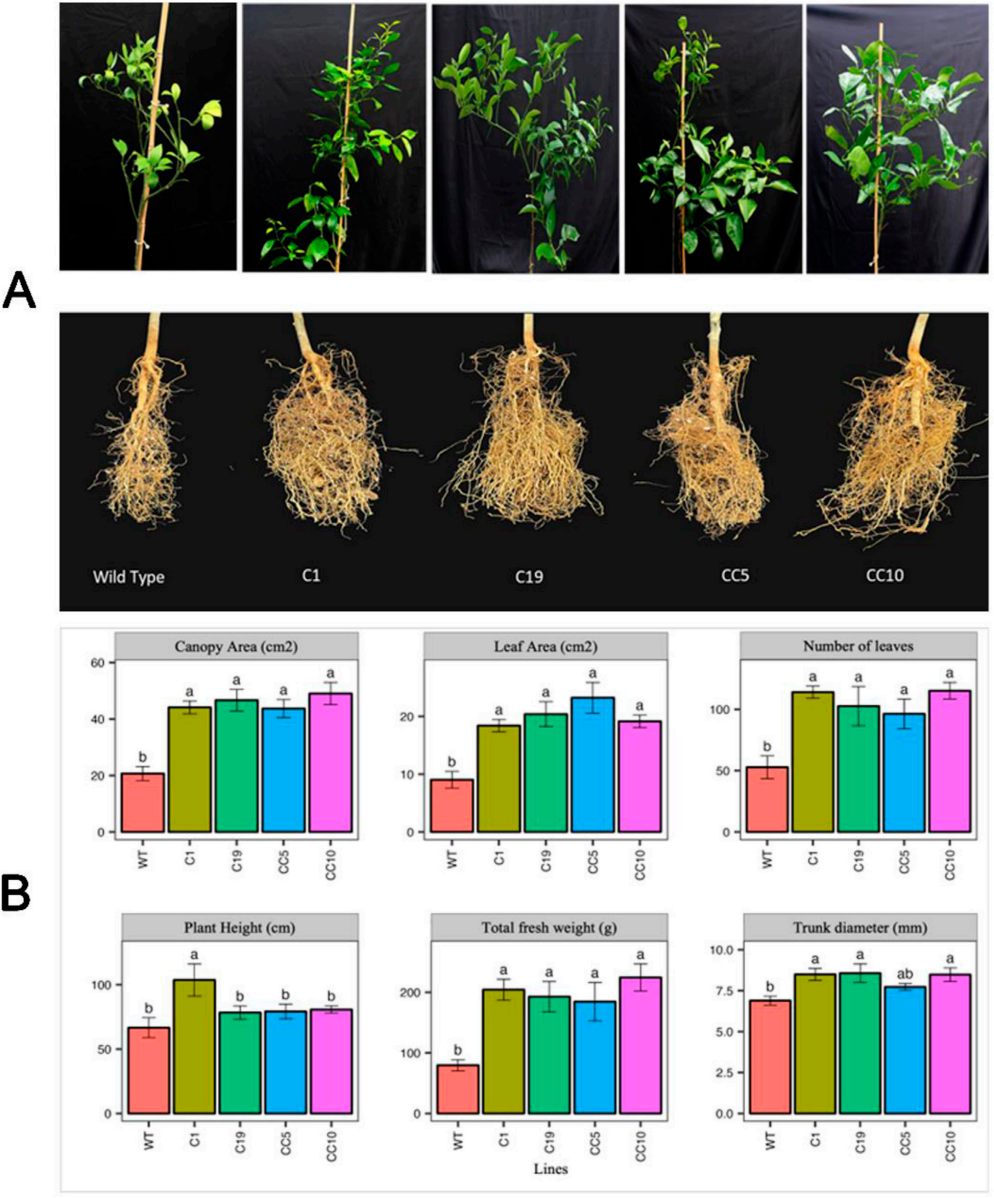
**(A)** Photos of the shoots and the roots of the edited and WT plants. Photos were taken after 24 months following infection. **(B)** Different growth parameters of *Ca*Las infected lines after 24 months of infection. One-way ANOVA was carried out for statistical analysis. Different letters on bars indicate statistically significant differences among lines by Fisher’s LSD at *p* < 0.05. Error bar indicates standard error. WT- Wild Type.

To assess various growth parameters of the infected trees, destructive sampling of the trees was performed at the end of the greenhouse study at 24 months after infection with *Ca*Las. The edited infected lines exhibited more leaves with larger leaf area, and greater trunk diameters compared to WT. Moreover, the edited trees exhibited higher fresh and dry weights than the WT trees. However, in terms of plant height, only line C1 displayed a significant increase compared to the other edited lines and WT (Figure 6).

### HLB altered the physiological parameters of the gene-edited trees

#### Chlorophyll content

**FIGURE 7:**
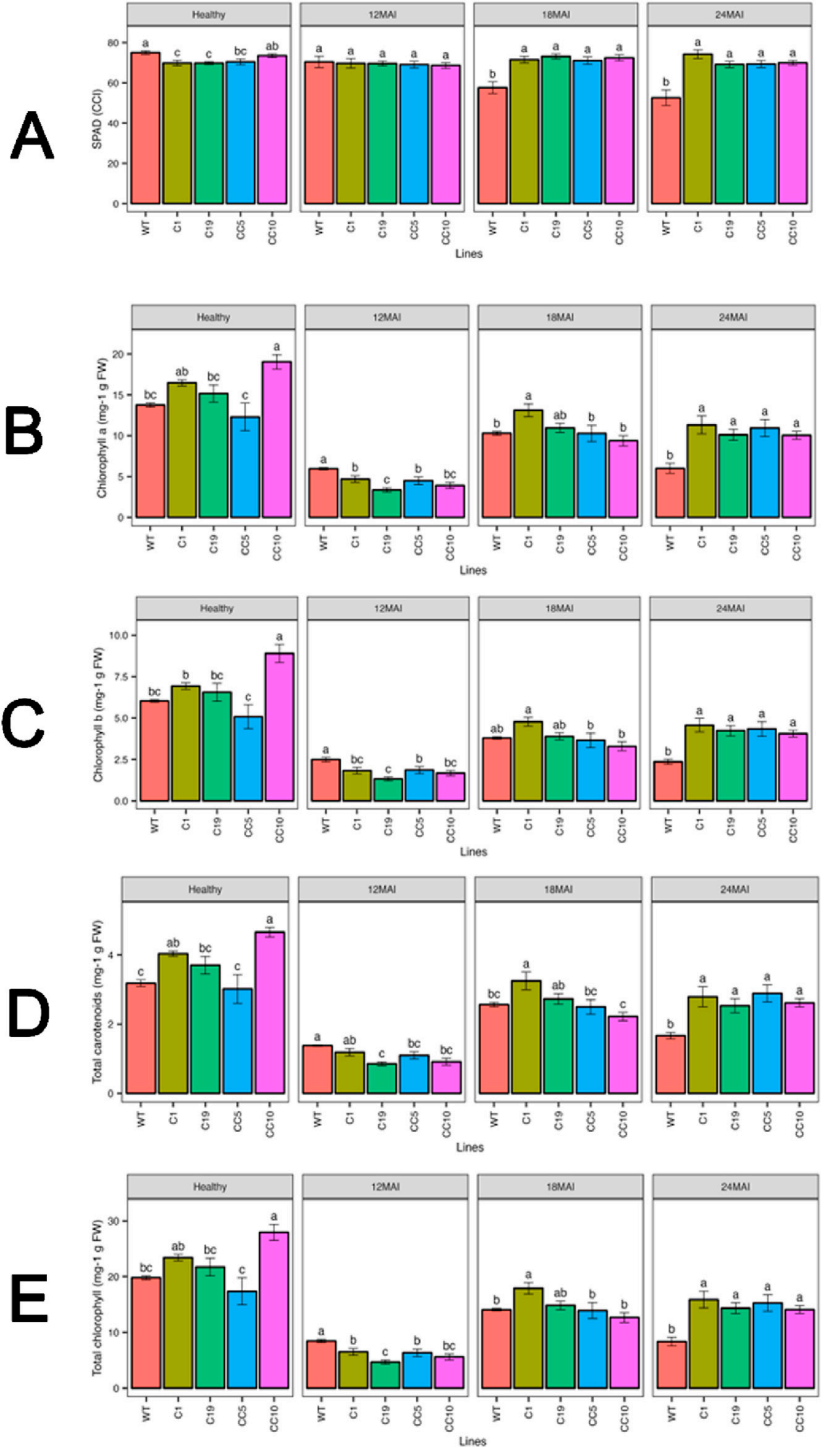
Chlorophyll content in the edited lines and control **(A)** SPAD measurements **(B)** Chlorophyll a **(C)** Chlorophyll b **(D)** Total carotenoids **(E)** Total chlorophyll. Analysis of Variance (ANOVA) was carried out separately for different times (12 MAI - 12 months after infection, 18 MAI- 18 months after infection, 24 MAI- 24 months after infection); WT- Wild Type. Different letters on bars indicate statistically significant differences among lines by Fisher’s LSD at *p* < 0.05). Error bar indicates standard error.

Before infection with *Ca*Las, the WT trees showed marginally higher chlorophyll content compared to lines C1 and C19. In healthy trees, line CC10 exhibited a notable increase in chlorophyll a, chlorophyll b, total carotenoids, and total chlorophyll content compared to the WT (Figure 7). In contrast, no significant differences were observed in the other edited lines compared to the WT. However, after 18 and 24 months of infection, the chlorophyll content was significantly higher in all edited lines compared to the WT trees. Similar trends were observed for chlorophyll b, total carotenoid, and total chlorophyll contents.

#### Starch content

**FIGURE 8:**
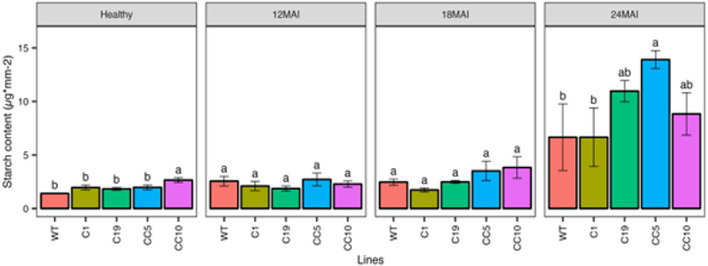
Starch content in the edited lines and control. Analysis of Variance (ANOVA) was carried out separately for different times (12 MAI - 12 months after infection, 18 MAI- 18 months after infection, 24 MAI- 24 months after infection); WT- Wild Type. Different letters on bars indicate statistically significant differences among lines by Fisher’s LSD at *p* < 0.05). Error bar indicates standard error.

After 24 months of infection with *Ca*Las, the edited lines showed higher starch content compared to the healthy trees (Figure 8). However, we found no significant difference between the starch content in the WT and edited lines at any other measured time point after infection.

#### Total phenolic content (TPC) and DPPH activity

**FIGURE 9:**
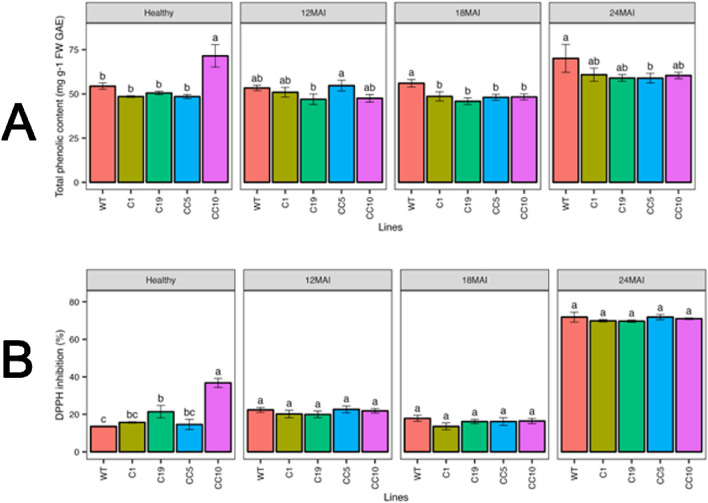
Physiological measurements in the edited lines and control. **(A)** Total phenolic compounds (TPC) and **(B)** DPPH activity. Analysis of Variance (ANOVA) was carried out separately for different times (12 MAI - 12 months after infection, 18 MAI- 18 months after infection, 24 MAI- 24 months after infection); WT- Wild Type. Different letters on bars indicate statistically significant differences among lines by Fisher’s LSD at *p* < 0.05). Error bar indicates standard error.

Line CC10 had the highest TPC in healthy trees, while other lines recorded similar TPC contents. However, in infected trees, TPC content varied at different sampling times. The TPC content ranged from 46.92 to 54.68 mg g^−1^ FW GAE at 12 months of infection, while it ranged from 45.8 to 56.02 mg g^−1^ FW GAE and 58.89–70.05 mg g^−1^ FW GAE at 18 and 24 months of infection, respectively. However, no significant changes in DPPH activity were observed between the healthy and infected lines (Figure 9).

#### *Ca*Las infection altered the transcript levels of several antioxidant related genes

**FIGURE 10:**
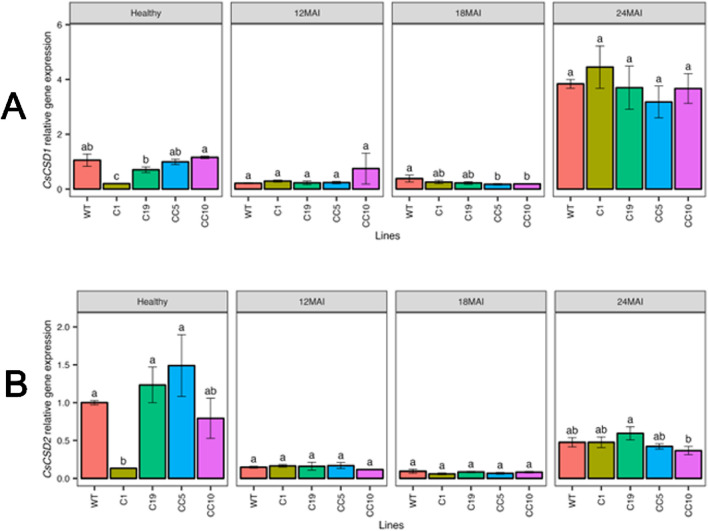
Relative gene expression level of *CsCSD1* **(A)** and *CsCSD2* **(B)** in the edited lines and wild type. Analysis of Variance (ANOVA) was carried out separately for different times (12 MAI - 12 months after infection, 18 MAI- 18 months after infection, 24 MAI- 24 months after infection); WT- Wild Type. Different letters on bars indicate statistically significant differences among lines by Fisher’s LSD at *p* < 0.05). Error bar indicates standard error.

Changes in the transcript levels of antioxidant genes related to plant defense were assessed in edited lines under healthy and infected conditions in comparison with WT trees (Figure 10). In healthy trees, the transcript levels of *CsCSD1* (Cu/ZnSOD1) and *CsCSD2* (Cu/ZnSOD2) decreased significantly only in line C1. However, all infected lines showed reduced transcript levels of *CsCSD1* and *CsCSD2* compared with those in healthy WT.

**FIGURE 11:**
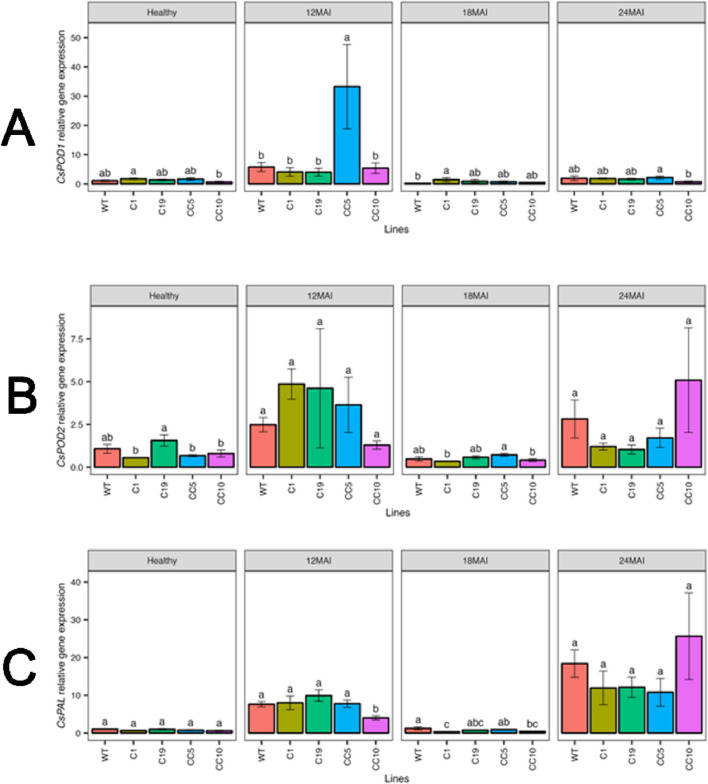
Relative gene expression level of *CsPOD1* **(A)**, *CsPOD2* **(B)** and *CsPAL* **(C)** in the edited lines and wild type. Analysis of Variance (ANOVA) was carried out separately for different times (12 MAI - 12 months after infection, 18 MAI- 18 months after infection, 24 MAI- 24 months after infection); WT- Wild Type. Different letters on bars indicate statistically significant differences among lines by Fisher’s LSD at *p* < 0.05). Error bar indicates standard error.

The transcript levels of *CsPOD1* and *CsPOD2* did not differ significantly among the edited and WT in healthy trees. No significant differences in *CsPOD1* and *CsPOD2* transcript levels were observed in the edited lines compared to those in the WT trees following *Ca*Las infection, except line CC5, which exhibited significantly upregulated *CsPOD1* transcript levels 12 months after infection. Under healthy or infected conditions, the *CsPAL* transcript levels did not differ significantly between the edited and WT trees (Figure 11).

**FIGURE 12:**
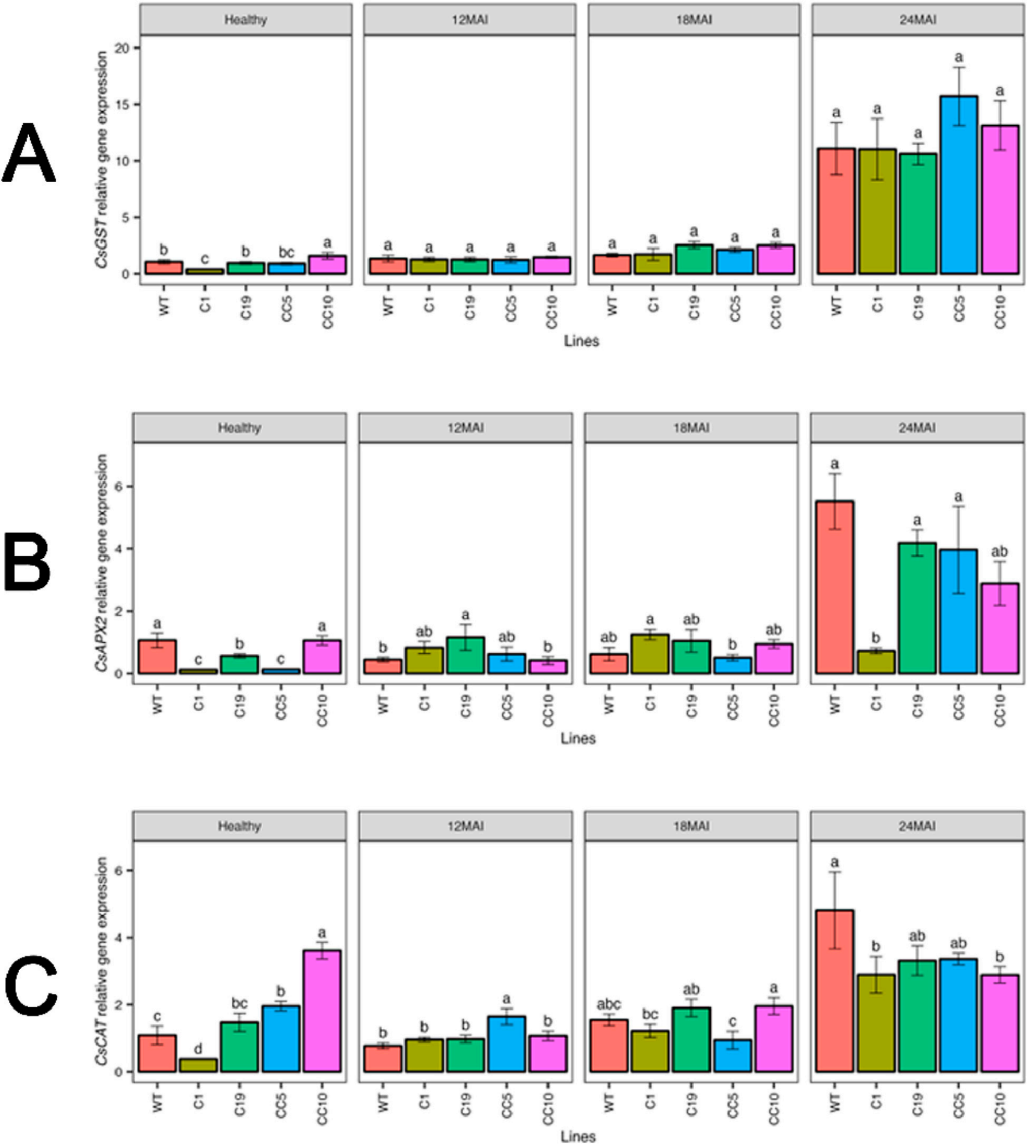
Relative gene expression level of *CsGST* **(A)**, *CsAPX2* **(B)** and *CsCAT* **(C)** in the edited lines and wild type. Analysis of Variance (ANOVA) was carried out separately for different times (12 MAI - 12 months after infection, 18 MAI- 18 months after infection, 24 MAI- 24 months after infection); WT- Wild Type. Different letters on bars indicate statistically significant differences among lines by Fisher’s LSD at *p* < 0.05). Error bar indicates standard error.

In healthy trees, *CsGST* transcript levels were elevated in CC10. Also by the end of the study, *CsGST* transcript levels increased significantly in infected trees compared with those in healthy WT trees. Similarly, under healthy conditions, CC10 exhibited a significantly higher transcript level of *CsCAT* than WT and other edited lines, while line C1 had the lowest transcript levels. After infection, notable differences in *CsCAT* transcript levels were seen in the edited lines compared to the wild type (WT). Although *CsCAT* expression was upregulated in all lines, there was no significant difference between the edited lines and the WT trees (Figure 12).

## Discussion

In this study, we employed a CRISPR/Cas9-based genome editing protocol to edit the *CsNPR3* gene in sweet orange and characterized the resulting trees’ morphological and physiological responses under greenhouse conditions following *Ca*Las infection. Genome editing using the CRISPR/Cas9 system can directly regulate the expression of endogenous genes at the transcriptional level (Lowder et al., 2015). We observed that editing of *CsNPR3* resulted in decreased to minimal gene expression (depending on the edited line) indicating the efficiency of the editing system. However, editing did not significantly affect *Ca*Las titers, indicating that *CsNPR3* silencing does not play a role in the infection process. However, since the morphological traits of the edited lines showed significant improvements in the infected edited lines, we can speculate that the overall tree health and the ability of the tree to respond following *Ca*Las infection is more important than just bacterial titers. The edited lines maintained better growth and vigor, despite being exposed to similar infection levels. Levy et al. (2023) proposed that HLB disease severity may be driven not only by the bacterial load but also by the plant’s own defense responses. Trees with enhanced stress tolerance, like the edited lines evaluated in this study, demonstrated improved performance under similar disease pressure. Therefore, knocking out *CsNPR3* appears to be a promising strategy to enhance tolerance to HLB disease, potentially boosting its ability to withstand the stress of HLB infection.

NPR3 and NPR1 have opposite roles during plant immunity (Liu et al., 2020). However, in our study, the *CsNPR1* gene was not highly upregulated in the non-infected trees, though its expression levels in line CC5 was significantly higher than in other lines. The expression levels of *NPR1*-associated genes, such as the *PR1, PR2*, and *PR5*, were low under non-stress conditions, indicating that these genes are less active or inactive in the absence of stressors (Qiu et al., 2020). Upon *Ca*Las infection we observed an increase in *CsNPR1* transcript levels. However, other *PR* genes were not consistently activated in these HLB-infected trees. This suggests that while *CsNPR3* editing may reduce the suppression of *CsNPR1*, and activate the SAR pathway, the complete activation of other *PR* genes downstream is not uniform across all the lines.

Several factors may account for these differences. In addition to *NPR3*, other genes, such as *NPR4*, negatively regulate the SAR mechanism in trees (Ali et al., 2024; Xu et al., 2019). Although the interaction of these genes is unclear in citrus, they likely influence the relative expression of PR genes seen across these lines. While *PR1* and *PR2* are known to trigger defense responses against different fungi and oomycetes, their specific roles against HLB still remain unknown (Hu et al., 2018). The lack of significant activation of *PR1* and *PR2* suggests that they might not be the primary drivers of defense against HLB under the conditions tested. However, the significant upregulation of *PR5* in some edited lines after infection highlights its potential role in stress responses.

*NDR1* acts as a mediator between ROS production and the subsequent production of SA in plant defense by facilitating the transmission of signals triggered by ROS to downstream constituents of the defense pathway (Shapiro and Zhang, 2001). In this study, we did not find significant expression of this gene in both healthy and infected trees compared to the respective WT trees. This was similar to the expression of other defense-related genes in the SAR pathway, suggesting a limited role in this context. However, the defense-related genes were not significantly upregulated, other physiological markers indicated improved health. We observed the higher levels of photosynthetic pigments after 24 months of infection that suggests a potential improvement in plant health by maintaining more efficient photosynthesis and thereby supporting the overall growth and health of the edited lines. Furthermore, we noticed that the free radical scavenging capacity and total phenolic content were increased after 24 months of *Ca*Las infection which aligns with the hypothesis that oxidative stress is playing a major role in managing stress.

We also observed that the edited trees demonstrated improved antioxidant responses, as indicated by the increased expression of genes such as *CsCAT* and *CsGST* in certain lines. Nehela and Killiny (2020) summarized three primary molecular mechanisms associated with HLB symptom development in citrus: (I) disruption in carbohydrate mechanism affecting the flow of nutrients and source–sink imbalance due to starch accumulation in leaves; (II) imbalance in stress-related phytohormones particularly the jasmonic and salicylic acid interaction; and (III) activation of detoxification proteins, specifically glutathione-S-transferases (GSTs), and regulation of antioxidant pathways. Our observation is consistent with the third proposed mechanism as we observed the increased expression of *CsCAT and CsGST*, which are involved in mitigating oxidative damage.

Different lines exhibited varying transcripts levels of the genes related to antioxidants. Under normal, unstressed conditions, the transcript levels of most antioxidant genes were relatively low, indicating that these genes are generally inactive without stress. However, the increased expression of *CsCAT* and *CsGST* in the CC10 line suggests that this plant experienced oxidative stress while the C1 line exhibited lower expression of all antioxidant genes than other lines, possibly indicating higher sensitivity to stress in this genotype. These differences in antioxidant defense mechanisms as well as other transcripts between the edited lines, likely resulted due to different gene insertion and deletion events during the gene editing process, resulting in inconsistent responses among the lines.

In *Arabidopsis*, *npr3 npr4* double mutants show increased disease resistance, accompanied by higher levels of *AtNPR1* and *PR* gene expression, compared to single mutants (Fu et al., 2012). Shi et al. (2013) reported enhanced disease resistance against the cacao pathogen *P. capsici* following the knockdown of the *TcNPR*3 gene. However, their findings were based on experiments conducted on detached leaves rather than on whole trees, which can overlook the temporal and systemic response of trees to stress over time. In contrast, our study examined four-year-old whole trees grown under greenhouse conditions that provides a more comprehensive understanding of how *CsNPR3* editing impacts the trees’ overall growth, defense mechanisms and tolerance for a longer period of time.

Taken together, the elevated expression of certain antioxidant genes in infected trees suggests that these trees were attempting to mitigate the effects of oxidative stress caused by the infection. However, the lack of significant changes in other antioxidant genes indicates that HLB may not exert a global impact on plant defense responses but rather a specific impact on oxidative stress regulation. Phenotypically, *Ca*Las-infected *CsNPR3*-edited trees exhibited greater vigor and fewer visible HLB symptoms compared to non-edited trees. This suggests that *Ca*Las titer alone may not be the sole determining factor influencing enhanced tree performance and eventual productivity, particularly under endemic HLB conditions.

## Conclusion

To our knowledge, this is the first study to analyze *CsNPR3*-edited citrus trees infected with *Ca*Las under greenhouse conditions. It provides insights into the complexity of developing tolerant varieties by editing a single gene in response to a complex disease like HLB in a woody tree. Our findings revealed that *Ca*Las-infected edited trees were more vigorous and exhibited fewer visible HLB symptoms compared to non-edited trees, despite the lack of significant differences in the expression levels of defense-related genes. The consistent phenolic content and percentage of DPPH inhibition suggest a minimal impact of the disease on these genome-edited trees, even following *Ca*Las infection. This suggests the possibility of other defense mechanisms at play, not explored in this study. The findings suggest that *CsNPR3* may not be the only factor that negatively regulates the SAR mechanism in citrus, highlighting the need to explore the effects of editing other homologs in addition to *CsNPR3*.

## Data Availability

The original contributions presented in the study are included in the article/Supplementary Material, further inquiries can be directed to the corresponding author.
