# Supplementary material for: Genetic and physiological characteristics of CsNPR3 edited citrus and their impact on HLB tolerance
Source: Front Genome Ed. 2024 Dec 4;6:1485529. doi: 10.3389/fgeed.2024.1485529 (PMC11652141; doi:10.3389/fgeed.2024.1485529)
Supplement: Supplementary file 1 [file DataSheet1.PDF]

Supplementary Material:

Supplementary Table 1. List of genes evaluated in this study.

Common Name	Forward primer	Reverse primer	Phytozome / NCBI Accession
CsNPR1	GTAGGCCGGCTGTTGATTT	GTCTAGGAGGTGCCTCTGATAA	orange1.1g007923m
CsNPR3	TCCTTGCTCAATGTGTTGATAGA	AAGACTTGAGTCGGAGCATTC	orange1.1g007849m
CsNPR4	AGAAAGTGGCTGGTGTATGG	CTCAGTCCTTTCCGACCTAATG	orange1.1g045501m
CsPR1	GTGGCGGAGAAAGCTAACTATAA	AACCCTAGCACATCCAACAC	orange1.1g048073m
CsPR2	ACAACCCAGTACGTGTCTTTC	TGCCGTGGAAACTTTGATTTG	orange1.1g019014m
CsPR5	CTCCGTTGTGGCTTGTAAGA	CTGTGTCGGAGAACACGTATC	orange1.1g026001m
CsSAM	GGACGCATCTTCTTGGGATAA	CGTGACAGTTTCCTTGACGA	XM_006466773
CsNDR1	TTCCTGCCCTCGACAAATC	CGTAGTAGACTCCCTTGTCTTTG	orange1.1.g028712m
CsCSD1	CAACTGTATCAGGAAGCCTCTC	CCAGTAGACATGCAACCATTTG	orange1.1g031837m
CsCSD2	CGCTCTTCCTCTTCTTCTTCTT	CGGCGAGAGATAAGTTGAGAC	orange1.1g026287m
CsAPX2	CCACATGGGTCTGAGTGATAAG	GTTAGTCCAGGGTCCTTCAAAT	orange1.1g025588m
CsPOD1	TTCGGAAGCGAATAGGGATATG	CCAAGAGTATGTCCACCTGATAAA	orange1.1g020635m
CsPOD2	ACAGGAAGAAGGGATGGTAGA	GACCAGGTCATGAACAGTAAGG	orange1.1g020619m
CsGST	GGCTTGACCAATTCAAACTACAC	GTTCATTGTCTCCTGGCTCTT	orange1.1g027134m
CsCAT	CTTCTTCTCCCATCATCCTGAAA	TCCTTCCATGTGCCTGTAATC	orange1.1g042356m
CsPAL	CTCGATGGCAGCTCTTATGTTA	GGTGAAGTTCTCAGGGCATAA	orange1.1g005031m
β-actin	GCTGCCTGATGGCCAGATC	AGTTGTAGGTAGTCTCATGAA	orange1.1g017124m

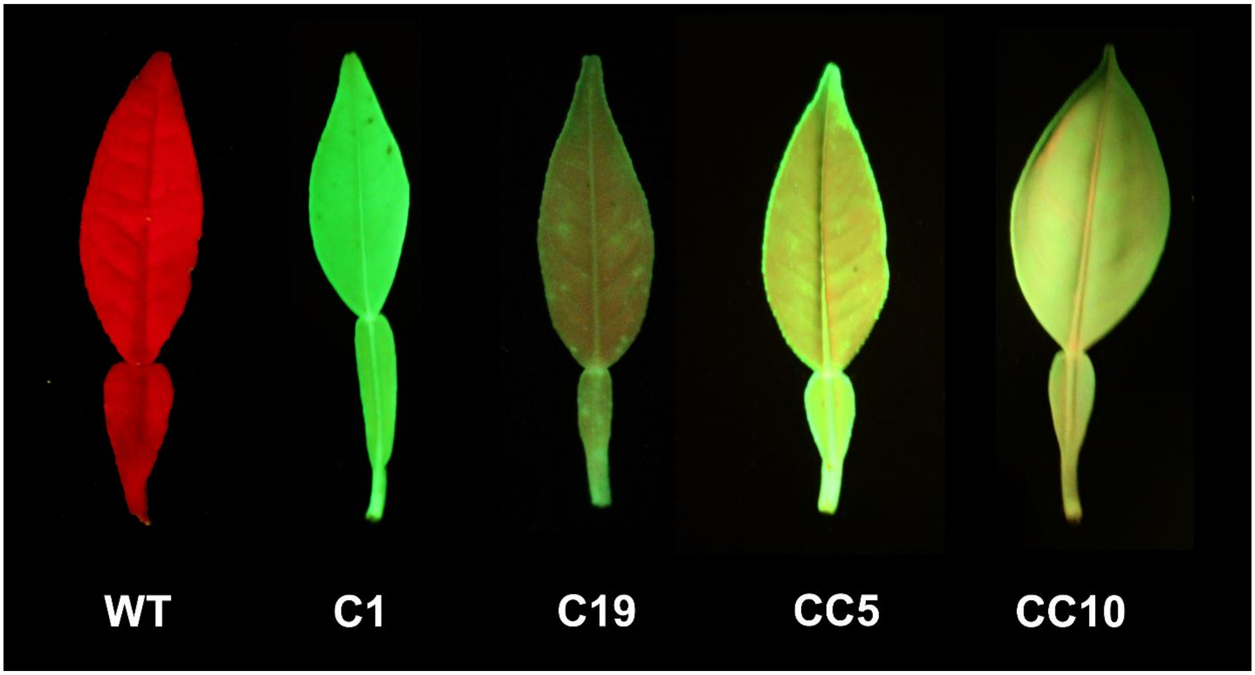

Supplementary Figure S1. GFP expression in young citrus leaves from the genome edited lines evaluated in the study. The green bandpass barrier filter of a NIGHTSEA Stereo Microscope Fluorescence Adapter was utilized to visualize and photograph leaves.
